# Accuracy of Conversion Disorder Diagnosis via Telestroke Network Consultation: A Retrospective Cohort Study

**DOI:** 10.31486/toj.25.0043

**Published:** 2025

**Authors:** Mugilan Poongkunran, Robin D. Ulep, Himanshu Chokhawala, Alaa E. Mohammed, Gabriel Vidal, Ifeanyi O. Iwuchukwu, Harold McGrade, Daniel Chehebar, Richard M. Zweifler

**Affiliations:** ^1^ Ochsner Neuroscience Institute, Ochsner Clinic Foundation, New Orleans, LA; ^2^ Department of Neurology, Icahn School of Medicine at Mount Sinai, New York, NY; ^3^ Department of Neurology, Ochsner LSU Health, Shreveport, LA; ^4^ Center for Outcomes Research, Ochsner Clinic Foundation, New Orleans, LA; ^5^ The University of Queensland Medical School, Ochsner Clinical School, New Orleans, LA

**Keywords:** *Conversion disorder*, *diagnostic errors*, *ischemic attack–transient*, *ischemic stroke*, *telemedicine*

## Abstract

**Background:**

Conversion disorder is a common mimic of ischemic stroke (IS) and transient ischemic attack (TIA). Data comparing the demographic and clinical features of patients with conversion disorder and IS/TIA are scarce, as are data evaluating the accuracy of conversion disorder diagnosis via telestroke consultation.

**Methods:**

We retrospectively analyzed consecutive patients evaluated through the Ochsner Health TeleStroke program from April 2015 through July 2016. Cases were classified into 2 categories: IS/TIA or conversion disorder. Patients with other stroke mimics and uncertain diagnoses were excluded. Both initial diagnosis and final diagnosis (following review of all records) were determined. The baseline characteristics of patients with conversion disorder were compared with the baseline characteristics of patients with IS/TIA. Initial diagnosis was compared with final diagnosis, and the diagnostic accuracy of telestroke consultations for conversion disorder was calculated.

**Results:**

We evaluated 885 patients with complete data. Of the 254 (28.7%) cases of stroke mimics, 50 cases (19.7%) were conversion disorder, representing 6% of all suspected strokes. We analyzed 616 patients with a final diagnosis of IS/TIA or conversion disorder. Compared to patients with IS/TIA, patients with conversion disorder were more likely to be female (*P*=0.0006) and younger (*P*<0.0001); were less likely to have diabetes mellitus (*P*=0.0252), hypertension (*P*=0.0004), or atrial fibrillation (*P*<0.0001); were less likely to receive tissue plasminogen activator (*P*<0.0001); and had a shorter median consultation duration (*P*=0.0175). The sensitivity, specificity, positive predictive value, and negative predictive value of conversion disorder diagnosis were 0.820, 0.998, 0.976, and 0.989, respectively. The adjusted area under the curve (95% CI) was 0.92 (0.87, 0.97).

**Conclusion:**

We found a conversion disorder rate of 19.7% of stroke mimics in the Ochsner Health TeleStroke Network, with a positive predictive value of 0.976 of discriminating conversion disorder from IS/TIA. In our study, patients with conversion disorder were more likely to be female and younger and to have fewer vascular risk factors compared with IS/TIA patients.

## INTRODUCTION

A stroke mimic is a condition that causes symptoms similar to a stroke but does not involve a blockage or bleeding in the brain. Examples include seizures, migraines, and fainting. Stroke mimics pose a serious challenge in the acute clinical diagnosis and management of stroke. Among any 3 or 4 patients seen by stroke teams, an estimated 1 patient does not have acute ischemic stroke (IS), resulting in substantial health and cost implications related to unnecessary hospital admissions, intravenous thrombolysis, and a higher level of care.^1-3^ As the burden of stroke increases, despite the reduction in age- and sex-adjusted incidence rates, we can expect the number of stroke mimics to increase substantially over the next decades.^4,5^

Patients with conversion disorder, also known as functional neurologic disorder, are a subgroup of patients admitted with a false-positive diagnosis of IS or transient ischemic attack (TIA). Although conversion disorder has been conventionally neglected as a less frequently occurring stroke mimic, interest in conversion disorder has grown as published evidence increasingly supports a neurobiologic role in the pathogenesis of functional disorder.^6,7^ A 2020 systematic review and meta-analysis by Jones et al reported a pooled prevalence of 15% for functional stroke mimics, with some studies reporting higher rates.^8^ However, most of the studies in the meta-analysis lacked descriptive data on functional disorders and lacked consistency in definitions, suggesting the possibility of reporting bias and underestimation.^8^ In another 2020 meta-analysis, Simhan et al reported that functional stroke mimics were more common in females and younger patients, corresponding with epidemiologic research on functional disorders.^8,9^ Recognition of conversion disorder remains a diagnostic challenge, however, especially in acute telestroke settings where the application of comprehensive examination techniques with the best predictive utility for the diagnosis of functional neurologic disorders is limited.^10^

Despite studies comparing stroke mimics with IS, a paucity of data is focused on telediagnosis and the comparison of clinical variables in patients with conversion disorder vs IS.^11^ We performed a retrospective analysis to determine the prevalence of conversion disorder in the Ochsner Health TeleStroke Network and to explore the characteristics differentiating patients with conversion disorder from patients with IS/TIA. We further sought to determine the diagnostic accuracy of conversion disorder in patients presenting with symptoms of acute cerebrovascular disease in our telestroke network. To our knowledge, our study is the first large-scale telestroke analysis comparing diagnostic accuracy metrics for conversion disorder vs IS/TIA.

## METHODS

After approval by the Ochsner Clinic Foundation Institutional Review Board, we retrospectively analyzed electronic health data from consecutive patients evaluated through the Ochsner Health TeleStroke program from April 2015 through July 2016. The Ochsner Health TeleStroke program is arranged in a hub-and-spoke configuration that includes the states of Louisiana, Mississippi, and Alabama. Ochsner Medical Center in New Orleans, Louisiana, serves as the hub where vascular neurologists are available around the clock via videoconferencing technology. For any patient with suspected acute stroke who presents within 12 hours of symptom onset (the activation window during the study period), emergency department (ED) physicians or licensed registered nurses in spoke hospitals have the ability to consult immediately with an Ochsner vascular neurologist. Initial diagnostic evaluation, neurovascular imaging recommendations and analysis, decision to treat with tissue plasminogen activator (tPA), and assessment of candidacy for mechanical thrombectomy are made following American Heart Association/American Stroke Association guidelines.^12^ The telestroke consultant completes all necessary documentation in the health system electronic medical record and coordinates care with the ED attending at the spoke hospital. During the study period, 36 spoke hospitals were in the network.

We identified 915 consecutive patients who were evaluated for suspected stroke. Information on demographics, baseline characteristics, clinical variables, diagnosis, and treatment were retrieved from the Ochsner Health TeleStroke database and electronic medical records. Data collected for the study included medical record number, age, sex, consultation duration in minutes, baseline National Institutes of Health Stroke Scale (NIHSS) score, vascular risk factors known at the time of presentation (eg, smoking, hypertension, diabetes mellitus, hyperlipidemia, coronary artery disease, atrial fibrillation, prior stroke/TIA), and tPA administered (yes/no).

After review of the primary vascular neurologist teleconsultation documentation, an independent investigator (RMZ) retrospectively confirmed the initial telestroke diagnosis and assigned the most likely diagnostic category. After review of all ancillary clinical and imaging data from the ED and hospital, an investigator (RDU) who was not involved in the initial telestroke evaluation retrospectively determined the final telestroke diagnosis. Reviewers classified a diagnosis as *uncertain* when lack of clarity precluded determining a diagnosis from the records. Patients whose hospital records were not available subsequent to telestroke consultation were not included in the analysis.

Consultations were classified into 1 of 2 diagnostic categories: IS/TIA or conversion disorder; other stroke mimics and uncertain diagnoses were excluded. For this study, we used the American Heart Association/American Stroke Association definitions for stroke and TIA.^13^ The vascular neurologist considered an initial telestroke diagnosis of conversion disorder when the presenting symptoms and/or clinical findings were incongruent with organic disease but the presentation was characterized by the physical diagnostic features observed in conversion disorder, normal neuroimaging results, the presence of psychiatric comorbidities, if any, and the exclusion of well-known stroke mimics. A final telestroke diagnosis of conversion disorder was made after review of all available records including local provider assessments and was determined to be consistent with criteria defined by *The Diagnostic and Statistical Manual of Mental Disorders*, 5th edition (DSM-5), as follows: (a) one or more symptoms of altered voluntary motor or sensory function are present; (b) clinical findings provide evidence of incompatibility between the symptom and recognized neurologic or medical conditions; (c) the symptom or deficit is not better explained by another medical or mental disorder; (d) the symptom or deficit causes clinically significant distress or impairment in social, occupational, or other important areas of functioning or warrants medical evaluation.^14^

Patient data are presented as median (interquartile range) for continuous variables and as counts and percentages for categorical variables. Bivariate analysis stratified by diagnostic category examined the association with age, sex, NIHSS score, vascular risk factors, tPA administration, and consultation duration. Statistical differences between the groups were assessed using the *t* test or Mann-Whitney *U* test for continuous variables and the Pearson chi-square test or Fisher exact test for categorical variables. We obtained summary estimates of sensitivity, specificity, positive predictive value, and negative predictive value for diagnosis of IS/TIA vs conversion disorder. The area under the receiver operating characteristic curve (AUC) was calculated to predict conversion disorder.

## RESULTS

Of the 915 Ochsner Health TeleStroke Network consultations conducted between April 2015 and July 2016, 885 patients had complete data for analysis. A total of 582 (65.8%) patients were diagnosed with stroke, 254 (28.7%) patients were diagnosed with stroke mimics, and 49 (5.5%) patients had an uncertain etiology at discharge. Among the 582 patients diagnosed with stroke, 460 were IS (79.0%), 106 were TIA (18.2%), 15 (2.6%) were intracerebral hemorrhage, and 1 (0.2%) was subdural hemorrhage.

Among the 254 patients diagnosed with stroke mimics, the most common noncerebrovascular etiologies were encephalopathy (n=63, 24.8%), conversion disorder (n=50, 19.7%), migraine (n=34, 13.4%), and seizures (n=33, 13.0%).

Given our focus on conversion disorder, we limited the study analysis to the 616 patients with a final diagnosis of IS/TIA (n=566) or conversion disorder (n=50). Patients with missing data, uncertain diagnosis, other stroke mimics, and intracranial and intracerebral hemorrhage were not included in the analysis.

The baseline demographic and clinical characteristics of patients with a final diagnosis of IS/TIA or conversion disorder are presented in Table 1. Patients in the conversion disorder group were younger than the patients in the IS/TIA group (*P*<0.0001), and more of the patients in the conversion disorder group were females (*P*=0.0006). Patients in the conversion disorder group had lower prevalences of the vascular risk factors hypertension, diabetes mellitus, and atrial fibrillation than patients diagnosed with IS/TIA. No statistically significant difference in the severity of presentation (NIHSS score) was found between the groups. Intravenous thrombolysis administration was more common in the IS/TIA group, and IS/TIA patients had longer consultation durations. Eight percent of the patients in the conversion disorder group received tPA, with none experiencing any fatal or nonfatal bleeding complication.

**Table 1.** Baseline Demographic and Clinical Characteristics of Patients with Ischemic Stroke/Transient Ischemic Attack vs Conversion Disorder, n=616

	Final Diagnosis		
Variable	Ischemic Stroke/Transient Ischemic Attack, n=566	Conversion Disorder, n=50	*P* Value
Age, years, median (Q1, Q3)	68 (58, 77)	46 (41, 53)	**<0.0001**
Female	278 (49.1)	37 (74.0)	**0.0006**
NIHSS score, median (Q1, Q3)	5 (2, 12)	4 (1, 9)	0.3308
Vascular risk factors			
Smoking	226 (39.9)	16 (32.0)	0.3074
Hypertension	476 (84.1)	31 (62.0)	**<0.0004**
Diabetes mellitus	249 (44.0)	14 (28.0)	**0.0252**
Hyperlipidemia	289 (51.1)	21 (42.0)	0.2185
Coronary artery disease	158 (27.9)	9 (18.0)	0.116
Atrial fibrillation	154 (27.2)	2 (4.0)	**<0.0001**
Prior stroke/transient ischemic attack	165 (29.2)	15 (30.0)	0.8997
Tissue plasminogen activator administered	190 (33.6)	4 (8.0)	**<0.0001**
Consultation duration, minutes, median (Q1, Q3)	17 (10, 25)	13 (7, 23)	**0.0175**

Notes: Data are presented as n (%) unless otherwise indicated. Statistically significant *P* values are set in bold text.

NIHSS, National Institutes of Health Stroke Scale; Q, quartile.

The conversion disorder diagnosis accuracy rate during the initial telestroke consultation was 97.6% (41/42) (Table 2). One patient with IS was misdiagnosed as conversion disorder during the initial telestroke consultation (false positive). Of the 50 patients with a final diagnosis of conversion disorder, 9 patients were not initially diagnosed correctly (false negative): 6 patients were initially diagnosed with IS, 1 patient was diagnosed with hypoglycemia, and 2 patients had an uncertain initial diagnosis. The sensitivity and specificity for diagnosing conversion disorder via telestroke consultation were high: sensitivity 0.820, negative predictive value 0.989, specificity 0.998, and positive predictive value 0.976. The adjusted AUC (95% CI) was 0.92 (0.87, 0.97).

**Table 2.** Accuracy of Initial Ochsner Health TeleStroke Network Diagnosis of Conversion Disorder

		Final Diagnosis		
		Conversion Disorder	Not Conversion Disorder	
**Initial Diagnosis**	**Conversion Disorder**	True positive, n=41	False positive, n=1	Positive predictive value=0.976
	**Not Conversion Disorder**	False negative, n=9	True negative, n=834	Negative predictive value=0.989
		Sensitivity=0.820	Specificity=0.998	

Note: All 885 patients with complete data were included in this analysis.

## DISCUSSION

In our study, we found that conversion disorder accounted for 19.7% of stroke mimics and represented 6% of all suspected strokes. The proportion of functional stroke mimics in our study is higher than previously reported estimates of <10%.^3,6,15^ The higher percentage of conversion disorder in our study may reflect the involvement of only stroke neurologists in establishing the diagnosis, consistency in diagnostic criteria and confirmatory test evaluation, and standardized care at the spoke hospitals, resulting in less exclusion rates. As noted earlier, a 2020 meta-analysis reported a pooled prevalence rate of 15% for functional stroke mimics when the definition of functional disorders was expanded to include depression and anxiety.^8^

The diagnostic accuracy of the Ochsner Health Telestroke Network for conversion disorder was high (98% positive predictive value). To our knowledge, our study is one of the first to report the diagnostic accuracy of conversion disorder in a telestroke network setting, supporting the utility of telestroke systems to accurately assess patients with acute functional neurologic presentations. In our previous analysis, we found excellent correlation between telestroke diagnosis and final discharge diagnosis for patients with both stroke and stroke mimics.^16^

Reliably differentiating stroke from conversion disorder is critical to avoid risks from unwarranted treatments; to limit excess direct and indirect expense to patients, hospitals, and payors; and to ensure patients receive the psychological care they need. Our bivariate analysis to identify the clinical predictors of IS/TIA vs conversion disorder showed that female sex and younger age were strong independent predictors of conversion disorder. Patients with conversion disorder were nearly 20 years younger, with a median age of 46 years, than the patients with IS/TIA and were approximately 1.5 times more likely to be female. Furthermore, we found significantly lower prevalences of the vascular risk factors hypertension, diabetes mellitus, and atrial fibrillation in patients with conversion disorder. The prevalence of smoking, hyperlipidemia, and coronary artery disease was also lower in patients with conversion disorder, but the lack of statistical significance possibly represents a type II error.

Other studies have reported similar findings, showing that patients with stroke mimics are younger and have fewer vascular risk factors than patients with strokes.^1,3,17^ However, prior studies of stroke mimics often combined conversion disorder with medical mimics,^1,2^ rather than evaluating conversion disorder specifically. Other reports comparing functional stroke mimics and medical stroke mimics have been from mixed, in-person settings rather than from vascular neurology telestroke programs where the accuracy of conversion disorder diagnosis may be higher.^6,8^ Our study, in which classification of functional mimics was based on evaluation by stroke neurologists, provides additional insights into IS/TIA vs conversion disorder in hyperacute settings.

We found no difference in NIHSS scores between patients with conversion disorder vs patients with IS/TIA. Even though some studies reported more weakness, sensory disturbances, and speech disturbances in patients with functional stroke mimics, no reliable symptoms or clinical signs consistently distinguish conversion disorder from stroke with precision.^18-21^ Although tPA was administered to 8% of patients with conversion disorder, none experienced bleeding complications. Previous analyses also showed no major adverse effects in patients with stroke mimics who received thrombolysis.^22-24^

Interestingly, the consultation duration was relatively brief in the conversion disorder group (median of 13 minutes in the conversion disorder group vs 17 minutes in the IS/TIA group). A possible explanation for the difference could be the statistically significant higher proportion of patients in the IS/TIA group who received tPA because longer consultation durations are required for cases involving administration of tPA.

Strengths of our study include the large, geographically diverse telestroke population and the 100% vascular neurology–driven model. Our cohort from 36 spoke hospitals included patients in the Stroke Belt with racial and socioeconomic diversity. Our study used a standardized categorization of stroke vs functional mimics that was based on stroke neurologist opinion and imaging results, thereby decreasing the bias created by inconsistencies in defining functional neurologic disorders.

Our study has limitations. Because the study is a telestroke-based review, our findings may not reflect the prevalence of stroke mimics in communities and clinics, limiting the generalizability to nonreferral hospitals. The retrospective design introduces the potential for measurement and observer bias. Limitations in data collection because of missing information and inaccuracies in data abstraction cannot be ruled out. In addition, because we focused the analysis only on clinical factors previously identified as predictive of stroke mimics, bias in measurement of risk factors, confounding variables, and outcomes is possible. Furthermore, physician diagnostic ascertainment bias with inter-rater variability may have occurred, especially for conversion disorder diagnosis, as we exclusively depended on the opinion of the treating stroke neurologist to classify patients, with no involvement of a neuropsychologist or neuropsychiatrist to further validate the diagnosis. Lack of neuropsychiatry inputs may have resulted in underdiagnosis of conversion disorder, particularly in rural spoke hospitals. We did not conduct a long-term follow-up evaluation, further limiting final diagnostic accuracy. The sample size of the conversion disorder group was relatively small, introducing the risk that some differences between both groups may have occurred by chance. Finally, the study cannot be generalized to hub-and-spoke models that offer expanded stroke care for a 24-hour window.

## CONCLUSION

In our patient population, we found a conversion disorder rate of 19.7% of all stroke mimics, with conversion disorder representing 6% of all suspected strokes evaluated within a large telestroke network. The positive predictive value of the Ochsner Health TeleStroke Network in discriminating IS/TIA and conversion disorder was 0.976. Patients with conversion disorder were more likely to be female, to be younger, and to have lower prevalences of vascular risk factors compared with IS/TIA patients. Patients with conversion disorder had shorter consultation times compared with IS/TIA patients, and tPA was administered less often in the conversion disorder group. An ideal supplement to our retrospective study would be prospective large-scale studies that explore the clinical predictors of conversion disorder and validate a predictive model. Such studies could also evaluate the cost implications of misdiagnosis and the long-term management of patients with conversion disorder.

## Notes

*This article meets the Accreditation Council for Graduate Medical Education and the American Board of Medical Specialties Maintenance of Certification competencies for Patient Care and Medical Knowledge.*
